# Development of Normal and Cleft Palate: A Central Role for Connective Tissue Growth Factor (CTGF)/CCN2

**DOI:** 10.3390/jdb6030018

**Published:** 2018-07-19

**Authors:** Joseph T. Tarr, Alex G. Lambi, James P. Bradley, Mary F. Barbe, Steven N. Popoff

**Affiliations:** 1 Department of Anatomy and Cell Biology, Lewis Katz School of Medicine at Temple University, Philadelphia, PA 19140, USA; joseph.tarr@temple.edu (J.T.T.); mbarbe@temple.edu (M.F.B.); 2 Division of Plastic and Reconstructive Surgery, David Geffen School of Medicine at UCLA, Los Angeles, CA 90095, USA; ALambi@mednet.ucla.edu; 3 Northwell Health Surgical Service Line, Department of Surgery, Zucker School of Medicine, Lake Success, NY 11042, USA; jbradley3@northwell.edu

**Keywords:** palatogenesis, cleft palate, connective tissue growth factor (CTGF/CCN2), bone morphogenetic proteins (BMPs), transforming growth factor-β (TGF-β), epidermal growth factor (EGF), fibroblast growth factors (FGFs), Wnt proteins

## Abstract

Development of the palate is the result of an organized series of events that require exquisite spatial and temporal regulation at the cellular level. There are a myriad of growth factors, receptors and signaling pathways that have been shown to play an important role in growth, elevation and/or fusion of the palatal shelves. Altered expression or activation of a number of these factors, receptors and signaling pathways have been shown to cause cleft palate in humans or mice with varying degrees of penetrance. This review will focus on connective tissue growth factor (CTGF) or CCN2, which was recently shown to play an essential role in formation of the secondary palate. Specifically, the absence of CCN2 in KO mice results in defective cellular processes that contribute to failure of palatal shelf growth, elevation and/or fusion. CCN2 is unique in that it has been shown to interact with a number of other factors important for palate development, including bone morphogenetic proteins (BMPs), fibroblast growth factors (FGFs), epidermal growth factor (EGF), Wnt proteins and transforming growth factor-βs (TGF-βs), thereby influencing their ability to bind to their receptors and mediate intracellular signaling. The role that these factors play in palate development and their specific interactions with CCN2 will also be reviewed. Future studies to elucidate the precise mechanisms of action for CCN2 and its interactions with other regulatory proteins during palatogenesis are expected to provide novel information with the potential for development of new pharmacologic or genetic treatment strategies for clinical intervention of cleft palate during development.

## 1. Introduction

Among previous reviews of palate development and cleft palate [1,2,3,4], the novelty of this review is its focus on the central role for CTGF/CCN2. This review begins with an overview of human and murine palatogenesis, the causes of orofacial clefting, and the anatomy of normal and cleft palate. We also briefly summarize the classification of cleft lip and palate and surgical repair of the cleft patient. We will then shift to a more in-depth review of the various factors, including growth factors, their receptors and/or signaling pathways that have been shown to play a role in the development of the normal palate. We emphasize the central role of connective tissue growth factor (CTGF), also known as CCN2, in regulating a number of important cellular events that are critical to palate development. CCN2 was first documented to play a role in palate development while characterizing its role in bone development [5]. Further studies showed more extensive involvement in craniofacial bone formation and altered underlying signaling [6,7]. We also discuss a number of other important factors that have been previously shown to regulate palate development as well as their interactions with CCN2. A brief discussion of future directions for research on the role of CCN2 in palatogenesis and orofacial clefting concludes this review.

### 1.1. Human and Murine Palatogenesis

**Figure 1:**
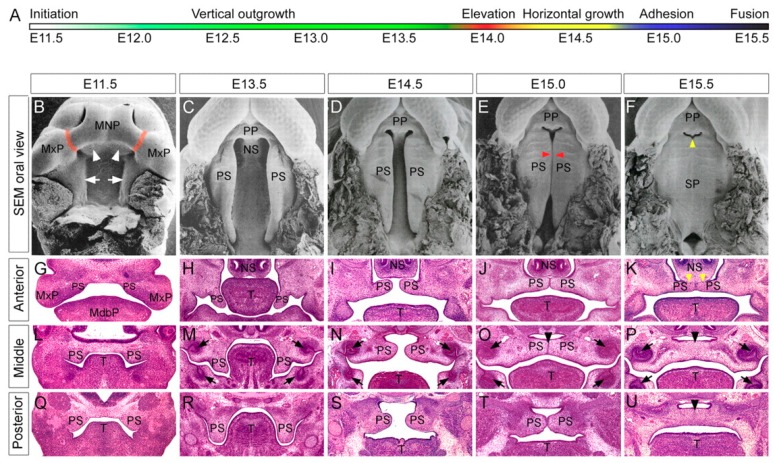
Normal mouse palatogenesis. Timeline of major developmental milestones in normal murine palatogenesis (**A**). Scanning electron microscopy of select developmental stages of murine palatogenesis as viewed from the oral cavity (**B**–**F**). Coronal H&E stained sections through anterior, middle, or posterior regions of the palate during selected developmental time points, E11.5 (**G**,**L**,**Q**), E13.5 (**H**,**M**,**R**), E14.5 (**I**,**N**,**S**), E15.0 (**J**,**O**,**T**), and E15.5 (**K**,**P**,**U**). MdbP, mandibular process; MNP, medial nasal process; MxP, maxillary process; NS, nasal septum; PP, primary palate; PS, palatal shelf; SP, secondary palate; T, tongue. (Reproduced/adapted with permission: Bush, J.; Jiang, R. Palatogenesis: morphogenetic and molecular mechanisms of secondary palate development. *Development* **2012**, *139*, 233, doi:10.1242/dev.067082).

The development of the palate occurs by identical processes in humans and mice. Formation of the palate occurs between the 4th and 12th weeks (W) of gestation in humans and between embryonic days (D) 11 and 15.5 in mice (Figure 1) [1,2]. The first step in development is the ventral (or ventrolateral) migration of neural crest cells from the neural folds of the developing brain to the mesodermal layer of the primordial face [1,2,3]. These neural crest cells contribute to the formation of mesenchyme. The face develops from five facial prominences: a (central) frontonasal prominence, and paired maxillary and mandibular prominences. The mesenchyme of the frontonasal prominence arises from neural crest cells from the midbrain and forebrain, while the maxillary and mandibular prominences arise from neural crest cells from the midbrain and hindbrain. Of note, the mandibular prominences develop from the first pharyngeal arch.

By the end of the 4th W in humans and D 9.5 in mice, these five facial swellings have formed around the primitive oral cavity, the stomodaeum [1,2,3,4]. During the 5th W, and D 10.0 in mice, ectoderm-derived nasal placodes form on the frontonasal prominence. As these enlarge through the 6th W, the center of the placode invaginates, thereby dividing the frontonasal prominence into medial and lateral nasal processes. These medial processes continue to grow throughout the 7th W (D 11.0 in mice) fusing to form the intermaxillary process—this will ultimately give rise to the philtrum and primary palate (anatomically distinguished from the secondary palate as anterior to the future incisive foramen) [4]. The upper lip and primary palate will complete formation, prior to initiation of secondary palatogenesis.

The secondary palate begins to form during the 7th W in humans and D 11 in mice. Proliferation of the neural crest cell-derived mesenchyme of the maxillary prominences results in a budding and development of primordial palatal shelves as medial extensions of the prominences [1,2]. These shelves are initially detectable in the early 7th W in humans and D 11.5 in mice [2]. Initially, the palatine shelves grow in a vertical direction, flanking the developing tongue [1,2]. After this initial period of shelf growth, the tongue flattens to assume its mature morphology, which clears the path for the vertically oriented palate shelves to elevate and assume a horizontal orientation. While the flattening of the tongue is considered to be necessary to prevent mechanical obstruction of shelf elevation, flattening alone is insufficient to cause elevation of the shelves [1]. The precise mechanism(s) responsible for shelf elevation are unknown. Shelf elevation occurs during the 7th-8th W of human and D 14.5 of mouse gestation [1,2,3]. Following elevation, the shelves continue to proliferate toward the midline, eventually making contact with each other at the medial edge epithelium [2,8]. This contact initiates a molecular cascade of factors that cause a combination of apoptosis and active extrusion of the epithelial cells at the midline until only a continuous band of mesenchymal cells remains [2,8]. Fusion begins at the incisive foramen and proceeds posteriorly in a zipper-like manner to terminate at the uvula [9]. Complete fusion occurs by the end of the 12th W in humans and D 17 in mice [2].

The secondary palate comprises of an anterior “hard” palate and a posterior “soft” palate. After midline fusion, mesenchymal condensations in the anterior portion of the secondary palate undergo intramembranous ossification from cranial neural crest derived-osteoblasts in the palatal mesenchyme [10,11,12]. Contact of these bony plates in the midline results in a mid-palate suture that remains patent into adolescence as the face continues to grow [10]. Posteriorly, mesenchymal condensations undergo myogenic differentiation and give rise to the musculature of the soft palate. Concurrent with secondary palatogenesis, the frontonasal prominence, and medial nasal processes continue to proliferate, forming a midline nasal septum [11,12]. This septum will grow caudally from the roof of the nasal cavity and fuse with the rostral surface of the primary and anterior secondary palates along the midline [11,12].

### 1.2. Causes of Facial Clefting

Abnormalities in any of the aforementioned steps of facial swelling development, growth, or fusion can result in orofacial clefting. Clefting may occur in a unilateral or bilateral fashion. It can also be syndromic (based on the presence of additional comorbidities) or non-syndromic [2,3,9]. The two most commons types of orofacial clefts are cleft lip and cleft palate. Cleft lip arises from deficient fusion of the mesenchyme of the maxillary prominence and intermaxillary process. The resulting cleft can range in phenotypic severity from a minor notch in the vermilion border of the lip to a complete separation of the lip from the philtrum and nasal cavity. Cleft palate results from failed midline fusion of the paired palatine shelves. Embryologic errors of formation leading to cleft palate include inadequate growth of the palatine shelves (e.g., failed neural crest cell migration), failed shelf elevation and fusion, and secondary degradation after fusion. Additionally, as proper palate formation requires mandibular growth with flattening and descent of the tongue, any aberrations in these processes can result in a cleft palate. 

Genetic predisposition for the development of facial defects combined with environmental factors can derail normal embryogenesis. To date, over 275 syndromes have been identified in which clefting is a primary clinical feature [13]. Specific genes involved include methylenetetrahydrofolate reductase (MTHFR) on chromosome 1, TGF-α on chromosome 2, MSX-1 on chromosome 4, BCL3 and TGF-β on chromosome 10, TGF-β3 on chromosome 14, and RAR-α on chromosome 17. Environmental factors linked to clefting include alcohol use, cigarette smoking, folic acid (and other nutritional) deficiencies, hypoxia, retinoid exposure, steroid use, and administration of anticonvulsants, such as phenobarbital and phenytoin [14].

### 1.3. Anatomy of the Normal and Cleft Human Palate

The normal palate is composed of the hard and soft palates and divides the oropharynx and nasopharynx. Both the oral and nasal surfaces are covered with a dense mucoperiosteum. The greater palatine neurovascular bundles emerge bilaterally from the palatine canals via the greater palatine foramina. While the hard palate continues the width and anterior projection of the maxillofacial framework, the soft palate functions as an active muscular valve—the velopharyngeal sphincter. Collectively, the muscles of the soft palate raise the sphincter toward the posterior pharyngeal wall, thereby separating the nose from the mouth. This function is critical for proper phonation, breathing, swallowing, and blowing.

The muscular soft palate, or velopharyngeal sphincter, is composed of five pairs of muscles: the levator veli palatini, tensor veli palatini, uvulae, palatopharyngeus muscles, and palatoglossus muscles [15]. Embryologically, the muscles of the palate are all derived from the fourth pharyngeal arch and therefore innervated by vagus nerve (pharyngeal branch). The sole exception to this is the tensor veli palatini, which is derived from the first pharyngeal arch, and therefore innervated by the mandibular division of the trigeminal nerve. The tensor and levator veli palatini muscles both arise from the Eustachian tube and are important anatomic components of cleft palate repair. The tensor veli palatini muscles arise from the medial pterygoid plate and spine of the sphenoid bone. They travel inferiorly as a tendon, coursing around the pterygoid hamulus, and insert on the soft palatal aponeurosis near the soft-hard palate junction. They function to control opening of the Eustachian tube, permitting aeration of the middle ear. The levator veli palatini muscles arise from the petrous portion of the temporal bone and from the cartilage of the Eustachian tube [15]. They travel inferomedially and insert at the midline on the palatine aponeurosis [15]. This interdigitating insertion forms the bulk of the anterior “levator sling” and therefore, the anterior portion of the velopharyngeal sphincter [15]. The uvulae originate from the nasal spine and palatine aponeurosis and insert on the mucosa of the uvula, assisting in elevation of the uvula [15]. The palatoglossus and palatopharyngeus muscles originate from palatine aponeurosis and course inferolaterally to insert on the lateral tongue and pharyngeal wall, respectively; both assist in narrowing the orophangeal aperture [15]. An additional pair of muscles are the superior pharyngeal constrictors; these originate from the medial pterygoid plate and Hamulus, pterygomandibular raphé, and mylohyoid line of the mandible, and course posteriorly to insert on the midline (median) pharyngeal raphe [15]. During velopharyngeal closure (e.g., swallowing), contraction causes anterior movement of a central (Passavant’s) ridge of the posterior pharynx to touch the posterior velum [15].

Anatomy of the cleft palate involves disruption of the levator sling and palatal aponeurosis, and normal muscular insertions thereon. Muscles such as the tensor and levator veli palatini, which would normally insert at the midline in a cleft, run longitudinally along the cleft margin before inserting into the posterior border of the hard palate. As a result, the normal sphincteric contraction of the soft palate and posterior pharynx is compromised. Incomplete closure results in air escape through the nose during the pronunciation of all but the nasal consonants and hypernasal speech. Additionally, as the tensor veli palatini assists with Eustachian tube opening, aberrant function likely contributes to the otopathology seen in cleft patients, including recurrent otitis media [14].

### 1.4. Classification of Cleft Lip and Palate

Owing to the diverse phenotypes of cleft lip and palate, multiple classification schemes have been described. Perhaps the most commonly clinically used classification system for cleft palate is from Victor Veau. Published in 1931, Veau simplified this system into four morphological forms: clefts of the soft palate (*divisions simples du voile*), clefts of the soft and hard palate (*divisions du voile et de la voûte*), clefts of the soft and hard palate extending unilaterally through the alveolus (*divisions du bec-de-lièvre unilatéral total*), and clefts of the soft and hard palate extending bilaterally through alveolus (*divisions du bec-de-lièvre bilatéral [total]*) [16]. Cleft lip deformities also encompass a spectrum of phenotypes. Veau himself outlined “varieties” for each of the labial defects, including laterality and completeness. These included examples such as complete unilateral cleft lip (*bec-de-lièvre unilatéral total sans division palatine*), complete bilateral cleft lip (*bec-de-lièvre bilatéral total sans division palatine*) [17]. Today, we typically describe cleft lip by its laterality and severity, the latter as mini-microform, microform, minor, incomplete, or complete [18,19].

An additional phenotype of clefting is the submucous cleft palate. Often difficult to diagnose as the palatal mucosa maintains visible continuity, the underlying muscular anatomy of the submucous cleft is similar to that of the classic cleft palate discussed above. Clinically, the phenotype is identified by Calnan’s classic triad, including a midline clear zone or zona pellucida (owing to the presence of the abutting oral and nasal mucosa without intervening musculature), a bifid uvula, and a palpable notch in the posterior hard palate [20].

### 1.5. General Approach to Surgical Repair of the Cleft Patient

The clinical approach to a patient with orofacial clefting is multidisciplinary. Because clefts can occur in concert with a clinical syndrome, appropriate workup and management of associate comorbidities is salient in early treatment of these patients. Children with isolated cleft palate suffer from feeding issues early in life as dysfunctional velopharyngeal sphincter precludes the closed seal require for suckling. The primary goals of cleft lip repair (cheiloplasty) are to recreate symmetry as well as realign the orbicularis oris muscle fibers to reproduce functional continuity. The goals of cleft palate repair (palatoplasty) are to produce complete closure of the palate and an intact and functioning velopharyngeal sphincter, most importantly before speech development. Owing to the great variety and severity of phenotypes of cleft lip and palate, various non-operative and operative strategies exist. For an overview of the surgical approaches to repair of cleft lip and palate, see van Aalst [14] and Monson [19].

### 1.6. Molecular Signaling in Normal Palatogenesis

At the time of palatogenesis, the palatal shelves exist as outgrowths from the maxillary tissue only a few mesenchymal cells in thickness and covered by a single cell layer of epithelium. The proteins that govern this process participate in a carefully choreographed ballet of interactions that are both temporally and spatially restricted. Each member of the molecular signaling cascade is required to be expressed at a precise time in the developmental timeline and at the precise location within the developing palate (reviewed by Bush et al., 2012) [2]. We will first focus on CTGF/CCN2, a relatively new factor to be shown to play an important role in palatogenesis. Global CCN2 knockout mice exhibit complete clefting of the secondary plate at 100% penetrance, and recent studies have shown that a number of the key cellular events necessary for normal palatogenesis are defective in the absence of CCN2. Furthermore, CCN2 can interact with a number of previously recognized molecular regulators of palatogenesis, and we will discuss their roles and the significance of their potential interactions with CCN2 during palate development. 

### 1.7. Connective Tissue Growth Factor

CTGF/CCN2 was originally described in 1991 by Grotendorst as a secreted platelet-derived growth factor (PDGF)-like factor derived from cultured human umbilical vein endothelial cells that competed with PDGF for binding to PDGF receptors on fibroblasts [21]. This new factor also displayed mitoattractant properties on cultured fibroblasts [21]. Further characterization of this protein elucidated that it was secreted by endothelial cells in addition to PDGF and could be detected with anti-PDGF antibodies [21]. Unlike dimeric PDGF, which has an apparent molecular mass of approximately 30 kDa, with a 17 kDa A chain (211 amino acids) and a 14 kDa B chain (241 amino acids), CTGF is comprised of 349 amino acids and has an apparent molecular mass of 38 kDa under both denaturing and reducing conditions, indicating that it is a monomeric protein [21]. Comparison of the primary amino acid sequence of CTGF and PDGF revealed homology of 40% indicating that although different in amino acid sequence, the detection of both CTGF and PDGF by the same antibody suggests that there are similar antigenic properties between the two proteins [21]. The human and mouse mRNAs are 81% identical and the proteins are 91% identical.

**Figure 2:**
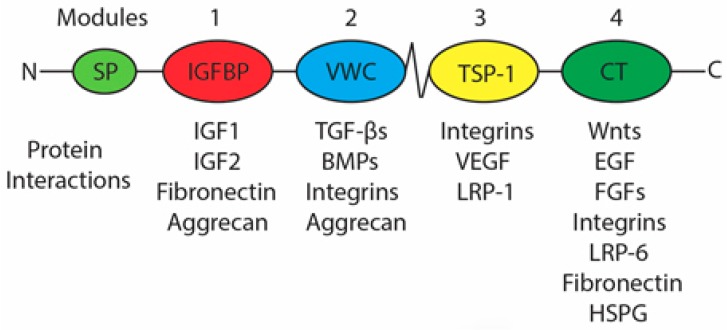
Structure and interaction of CCN Family Member Modules. Diagrammatic illustration of the domain structure of CCN proteins highlighting the signal peptide (SP), 4 active modules (IGFBP, VWC, TSP-1, and CT) and known protein interactions specific to each module [23,24,25,26,27].

CTGF is the second member of the CCN protein family and is now formally referred to as CCN2 [22]. The CCN family of proteins was named for its first three members, cysteine rich angiogenic inducer 61 (CYR61/CCN1), connective tissue growth factor (CTGF/CCN2) and nephroblastoma overexpressed (NOV/CCN3) with each member being numbered based on order of discovery [22,23]. Later, the family was expanded to contain the Wnt-induced secreted proteins 1 (WISP1/CCN4), 2 (WISP2/CCN5), and 3 (WISP3/CCN6) [22,23]. Each member of the CCN protein family possesses the same structure, a signal peptide plus 4 active modules (Figure 2) with the exception of CCN5 which does not contain the 4th module [23]. The 4 modules include an insulin growth factor binding protein domain (IGFBP), a von Willebrand type C domain (VWC), a thrombospondin-1 domain (TSP-1), and a cysteine knot containing C-terminal domain (CT) (Figure 2) [23]. A unique characteristic of the CCN protein family is that, to date, no unique receptor has been identified that is responsible for the actions of the CCN proteins [24]. Therefore, it is hypothesized that the actions associated with the CCN proteins are due to the binding properties that each module possesses and the specific interactions that modulate the function of other proteins with which they interact.

CCN2 is a secreted protein that becomes incorporated into and influences the interactions of cells with the extracellular matrix (ECM) but does not contribute to the structural properties of the ECM; thus, CCN2 is termed a matricellular protein [21,23]. Thus, many of the protein-CCN2 interactions exist with proteins known to impact the ECM such as TGF-βs, BMPs, FGFs, EGF, and various integrins (Figure 2) [26,28,29,30,31,32,33,34]. The four modules that compose CCN2 have distinct biological activities and the different modules retain biological activity when cleaved apart from the full-length CCN2 protein [35,36,37]. Matrix metalloproteinases (MMPs) appear to be the family of proteases responsible for CCN protein module cleavage [35,36].

CCN2 is involved in a multitude of functions in both normal and pathological conditions. Among the most-studied functions of CCN2 are its role in angiogenesis, chondrogenesis, and skeletogenesis/fracture healing [5,6,23,28,30,31]. In these processes, CCN2 promotes cellular proliferation, survival, adhesion, migration, differentiation, and ECM production [5,6,23,26,28,30,38]. Given these functions of CCN2, in pathological states where the expression of CCN2 is elevated, CCN2 expression is commonly associated with increased fibrotic responses and chemotherapeutic-resistant metastatic cancers [39,40,41,42,43,44,45].

#### 1.7.1. Regulation of CCN2 Expression

According to the National Center for Biotechnology Information (NCBI), the gene for CCN2 resides on human chromosome 6 at position 6q23.2 and chromosome 10 in the mouse. It contains 5 exons encoding the signal peptide and the 4 active modules as described above. The best-known signaling pathway that governs CCN2 expression is the TGF-β1 pathway [46]. Other, less well-characterized and more-cell specific regulators of CCN2 expression are yes associated protein (YAP), BMPs, substance P (SP), vascular endothelial growth factor (VEGF), and Wnt-proteins [47,48,49,50,51,52].

TGF-β1 influences CCN2 gene transcription through two downstream effectors, the Smad2/3/4 complex, and extracellular regulated kinase 1/2 (ERK1/2) [46]. TGF-β1 ligand first must bind to the TGF-β type 1 receptor (TGFβ-R1), also known as activin receptor-like kinase 5 (ALK5) [53]. Next, the type 1 receptor dimerizes with TGF-β type II receptor (TGFβ-R2) forming the active receptor [53,54]. Both TGFβ-R1 and TGFβ-R2 are transmembrane serine/threonine kinases. There also exists a TGFβ-R3 that does not possess cytoplasmic signaling function, but has recently been reported to be necessary in activation of other downstream TGF-β signaling pathways [55,56]. TGFβ-R3 acts as an affinity enhancer of the TGF-β ligand resulting in increased signaling of the dimeric TGFβ-R1/TGFβ-R2 complex [54]. To accomplish this, TGFβ-R3 forms a transient heterodimeric complex with TGFβ-R2, thereby increasing the affinity of TGFβ-R2 for TGF-β ligands [55]. Subsequent binding of TGF-β ligands to TGFβ-R2 causes a rapid dimerization with TGFβ-R1 and simultaneous displacement of TGFβ-R3, leading to normal receptor signaling [55]. Upon dimerization between the type 1 and type 2 TGF-β receptors, the TGFβ-R2 phosphorylates the TGFβ-R1’s cytoplasmic region resulting in initiation of the signaling cascade [53,54]. This interaction between the two subunits of the receptor limits signaling exclusively to the dimeric form of the receptor.

While several signaling pathways are activated in TGF-β1 signaling, the canonical Smad or non-canonical mitogen-activated protein kinase (MAPK) pathways are the two that regulate CCN2 gene transcription [46,53]. Following activation of the TGFβ-Rs at the cell membrane, the serine/threonine kinase phosphorylates Smad2 and Smad3 (receptor-regulated Smads [R-Smads]), in the cytoplasm [46,53]. Phosphorylation of Smads 2 and 3 occurs at two C-terminal serine residues and results in an acidic knob that binds to other R-Smad C-terminal acidic knobs and to Smad4 [53]. The trimeric complex formed between Smad2/3 and Smad4 allows for translocation of the Smad2/3/4 trimer into the nucleus [46]. Smad4 also binds to other R-Smads and thus is termed a common Smad [30,46,53]. Regulation of this process is achieved through Smad7, an inhibitory Smad protein that prevents phosphorylation of R-Smads and/or prevents nuclear translocation of the Smad2/3/4 trimers [46]. Once in the nucleus, the DNA binding hairpin structure found in R-Smads allows binding to the Smad binding elements (SBEs), DNA sequence CAGAC, and thereby enhances transcription of CCN2 mRNA by increasing gene transcription [46,53]. Increased transcription is achieved through recruitment of p300 and cAMP-response element binding protein (CREB), histone acetyl transferases that together relax the chromatin structure in the promoter to enhance gene expression [46,53]. P300 and CREB act as adapter molecules to promote assembly of the preinitiation complex, including RNA polymerase II [46,53]. Smads 1, 2, 3, and 5 all share identical primary structure and recognize the same SBE; thus, it is the combination of Smads within the nuclear complex that allows for differential recognition of SBEs in the promoter region of target genes [53].

TGF-β1 signaling through the receptor complex also activates several members of the MAPK protein family, including p38, JNK1/2, and ERK1/2 [46]. Stimulation with TGF-β1 in combination with an ERK1/2 inhibitor or dominant negative form of ERK1/2 inhibits CCN2 expression suggesting that only ERK1/2 is crucial for TGF-β1 mediated CCN2 transcription [46]. Inhibition of Src kinase activity was found to eliminate upregulation of CCN2 following TGF-β1 treatment demonstrating that this nonreceptor tyrosine kinase also plays an essential role in transcriptional regulation of CCN2 by TGF-β1 [46].

While TGF-β1 is the most-studied stimulator of CCN2 expression, other proteins have also been shown to regulate CCN2 expression [47,48,49,50,51,52]. Yes associated protein (YAP), a downstream mediator of the Hippo kinase cascade, is known to influence CCN2 transcription through complex formation with the transcriptional enhancer associate domain (TEAD) transcription factor [49]. Only the TEAD transcription factor recognizes the binding sequence GGAATG within the promoter region of CCN2; however without YAP, the TEAD factor is unable to initiate CCN2 transcription [49]. BMPs, like TGF-β1, are believed to influence CCN2 transcription via R-Smads, specifically Smad1/5/8 though non-canonical pathways may also play an important role in regulating CCN2 transcription [52,57,58]. While many BMPs exist, currently only BMP2, BMP6, and BMP9 have been clearly demonstrated to influence CCN2 transcript levels, though not as effectively as TGF-β1 [52,58]. Substance P (SP) has recently been described as being able to increase CCN2 protein production through increasing TGF-β1 levels in tenocytes [59]. VEGF is capable of increasing CCN2 protein in endothelial cells similar to TGF-β1 through a PI3k-Akt dependent mechanism that is Ras and ERK independent [51]. This mechanism is likely the underlying mechanism behind reports that hypoxic environments also increase CCN2 production and logically links the angiogenic effects of CCN2 with the hypoxic drive to produce new blood vessels. Finally, Wnt proteins have been described as influencing CCN2 expression but the mechanism remains unclear. Wnt3a appears to influence CCN2 expression in C3H10T1/2 cells through a β-catenin dependent mechanism although specific details on how this pathway increases CCN2 production are currently unknown [52].

#### 1.7.2. Role of CCN2 in Palatogenesis

Model organisms are crucial to the study of genes of interest in both physiological and pathological settings. Two global CCN2 knockout (KO) models currently exist; one is a complete knockout in which no portion of CCN2 is expressed, while the other model retains expression of the signal peptide and the first active module [5,60]. Extensive phenotypic analysis reveals that despite these differences, the two models are remarkably similar [5,60]. Although CCN2 KO animals appear to die of hypoxia within minutes after birth, they do complete the embryonic developmental process [5,7]. Other characteristic defects observed in both models include lung hypoplasia, various craniofacial morphological changes, reduced chondrocyte proliferation in long bone growth plates, and distinct kinking of the ribs, radii, ulnae, and tibias [5,6,60]. CCN2 heterozygous mice do not exhibit any of these defects; they develop and grow normally, and are capable of normal rates of reproduction.

The craniofacial anomalies of the CCN2 KO include decreased bone mass of the mandible and parietal bones, while having a decreased skull length with increased skull width [2]. This study also noted that midline facial structures were abnormal in the CCN2 KO mice with regards to the vomer, maxillary and palatine processes failing to meet at the midline, resulting in anomalous nasal cavity architecture [6]. The failure of the palatine bones to meet and fuse at the midline reinforced the initial documentation of orofacial clefting in the CCN2 KO model [5,6]. Although these studies of CCN2 KO mice alluded to a cleft palate phenotype, the mechanisms were unknown [5,6]. A more recent study utilizing CCN2 KO mice and cranial neural crest-derived mesenchymal cells from these mice provided unequivocal evidence for the vital role of CCN2 in palate development [7]. The three processes that are essential for normal palate development, namely growth (proliferation), elevation and fusion of the palatal shelves, are all negatively affected in CCN2 KO mice [7]. The absence of CCN2 inhibits the proliferation of palate mesenchymal cells specifically at the G1/S transition [7]. Absence of CCN2 also inhibits elevation of the palatal shelves from the vertical to horizontal position [7]. CCN2 KO mesenchymal cells demonstrated deficiencies in cell adhesion and spreading related to an inability to activate Rac1 and RhoA [7]. Contrary to these defects in cell function, CCN2 KO mesenchymal cells exhibit increased migration rates compared to WT cells [7]. Addition of exogenous CCN2 was able to restore the adhesion and spreading of CCN2 KO cells to normal levels, but was ineffective in altering the defective proliferation of these cells [7]. Using an organ culture model, it was also shown that the palatal shelves from CCN2 KO mice failed to fuse as indicated by the retention of the medial edge epithelium [7]. Even when the palatal shelves were apposed to one another, the mechanisms governing fusion were aberrant in the absence of CCN2.

### 1.8. Proteins that Regulate Palatogenesis and Their Interactions with CCN2

CCN2 impacts the function of many proteins through the varied interactions of its four modules and likewise the expression of CCN2 is controlled by a number of factors. Mammalian palatogenesis is also controlled by a myriad of factors that are exquisitely temporally and spatially regulated in the developing palate. Thus, it is not surprising that CCN2 interacts with many of the proteins that govern palatogenesis, including BMPs, FGFs, EGF, Wnts, and TGF-βs [24,29,30,52,61,62]. 

#### 1.8.1. Bone Morphogenetic Proteins (BMPs)

BMPs play roles in a multitude of normal and pathological processes, including embryonic development, fracture healing, and cancer [63]. Clinically, BMPs are used in the treatment of non-union fractures, osteoporosis, and calvaria defects [63,64]. BMPs are members of the TGF-β superfamily of proteins and, like TGF-βs, signal through complexes of receptor serine/threonine kinases [30,63,64,65]. Like TGF-βs, the functional signaling complex for BMPs is a ligand dimer and a tetrameric receptor complex composed of two type 1 and two type 2 receptors, where the type 2 receptors phosphorylate the type 1 receptors upon ligand binding to initiate cytoplasmic signaling cascades, including the MAPKs and R-Smads 1, 5, and 8 [64,65].

**Figure 3:**
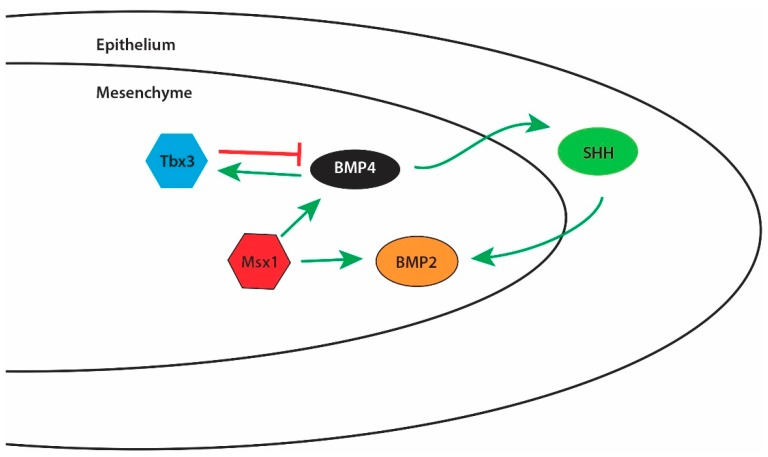
A simplified schematic of Tbx3, BMP4, SHH, and BMP2 signaling in the regulation of proliferation in the developing palate. Within the mesenchymal tissue of the developing palate, Tbx3 and BMP4 co-exist in a regulatory loop that governs both Tbx3 and BMP4 expression levels. BMP4 is able to cross into the epithelial cell layer to influence SHH expression within the epithelium. SHH is then capable of crossing back into the mesenchymal tissue to influence BMP2 expression. BMP4 and BMP2 expression may also be controlled independently through Msx1 activity in the mesenchymal tissue of the developing palate.

There are over 20 BMP family ligands, yet in palate development only BMP2, BMP4, and BMP7 have been shown to play a significant role [9,65,66]. Likewise, there are many variants of the BMP receptor, yet to date only BMPR1a appears to be important in regulating the molecular processes governing normal palate development [9,67]. BMP2 and BMP4 appear to be primarily involved in governing proliferation of the developing palatal mesenchyme [9,65,66]. Upstream of both BMP2 and BMP4 is the homeobox transcription factor Msx1 [9,66]. Msx1 regulates the transcription of BMP4 in the anterior region of the developing palate [9,68]. BMP4 is diffusely expressed through the palatal shelf at E13.5 in mouse development and then becomes restricted to the mesenchymal layer immediately beneath the epithelium on the oral side of the shelf by E14.5 [68]. To control the rate of mesenchymal cell proliferation in the palate, BMP4 is also regulated by the transcription factor Tbx3 [68]. Tbx3 inhibits expression of BMP4 while the expression of BMP4 induces expression of Tbx3 [68]. This feedback loop is believed to be one of several such feedback loops controlling the expression of BMP4 and BMP4-mediated cell proliferation and migration in the developing palate mesenchyme, as knockout of Tbx3 does not cause cleft palate in mouse models [9,68]. BMP4 is also known to induce sonic hedgehog (SHH) expression in the oral side epithelium of the developing palate, specifically at the rugae [9,66,69]. Expression of SHH is limited to the rugae of the oral epithelium throughout palate development [70,71]. SHH then may signal back into the mesenchymal tissue to induce mesenchymal cell proliferation via increased BMP2 expression [9,66,69] (Figure 3).

Figure 3 depicts some well-documented interactions among a select number of signaling molecules involved in the regulation of mesenchymal cell proliferation during palatogenesis. However, a decrease in both BMP4 and SHH signaling in the developing palate at E13.5 results in little change in BMP2 expression in the anterior portion of the palate, thereby suggesting that BMP2 is regulated by additional pathways that are distinct between the anterior and posterior parts of the developing palate and which have yet to be elucidated [72]. BMP7 expression is also regulated by Msx1 but exists in a regulatory feedback loop with its antagonist follistatin; restricted to the nasal side epithelium in the developing palatal shelves [73]. Follistatin and BMP7 expression are restricted to an area of the developing palate that is distinct and non-overlapping with BMP2 and BMP4 [73]. The BMP receptor primarily involved in regulating proliferation in the developing palate is BMPR1a, located in the anterior portion of the palate [74]. The global knockout of BMPR1a is embryonic lethal at the gastrulation stage and thus conditional knockout Wnt1-Cre/BMPR1a mice are commonly used to studying the role of BMPR1a in palatogenesis [67,74]. These mice show 100% penetrance of clefting within the anterior portions of the secondary palate as a result of decreased proliferation of the palate mesenchymal cells [67,74].

Modulation of ligand-receptor binding and receptor activity is a hallmark function of the CCN protein family. CCN2 is well documented to inhibit the signaling activity of BMP2 and 4 by negatively modulating binding of BMPs to the BMPR [30]. CCN2 reduces total Smad1/5/8 levels and phosphorylated Smad1/5/8 levels secondary to BMP4 and BMP2 treatment of cultured mesodermal stem cells or calvaria-derived osteoblasts, respectively, by interacting with the ligand and inhibiting its affinity for the receptor [29,30,75]. In chondrocytes, in addition to the negative impact on Smad1/5/8 signaling, CCN2 is also known to inhibit ERK1/2 phosphorylation, but not total ERK1/2 protein levels, following BMP2 stimulation [75]. Additionally, CCN2 has been shown to inhibit the upregulation of genes controlled by BMP2 signaling in skeletogenesis, including Runx2, alkaline phosphatase, and type I collagena1 [30]. CCN2 therefore appears to be acting as a vital negative regulator of BMP activity in many regions of the developing body and may also function in this capacity in the developing palate. The inhibitory effects that CCN2 plays on BMP could result in enhancement of these processes in the absence of CCN2, and this may be the case with cell migration, which is increased in KO compared to WT palate mesenchymal cells.

#### 1.8.2. Fibroblast Growth Factors (FGFs)

FGFs are involved in the early stages of embryonic development, tissue repair and maintenance, and metabolism regulation [76,77]. There are currently 22 known human FGF ligands that are further subdivided into 7 subfamilies based on function and binding affinity to the 5 known FGF receptors [78]. FGF receptors (FGFRs) are receptor tyrosine kinases and, like BMPs and TGF-βs, signal only as functional dimers [77]. The extracellular domains of the FGF receptors are composed of 3 Immunoglobulin (Ig)-like domains existing in series in which the Ig I domain is believed to inhibit ligand binding and in which II and III regulate ligand specificity [77,78]. FGFR 1, 2 and 3 may be further subdivided into IIIb and IIIc variants based on alternative splicing of the 2nd half of the 3rd Ig domain to further regulate ligand specificity [77,78]. Generally, FGFRb isoforms exist in the epithelium and bind mesenchymally expressed FGF ligands while FGFRc isoforms are the inverse [79,80]. FGFR4 does not undergo this alternative splicing and the FGFR5 lacks intracellular signaling domains and is believed to play an inhibitory role in ligand binding with other receptors to further regulate FGF activity [76,77]. Activation of FGFR signaling initiates a cytoplasmic signaling cascade that may result in an increase in cytoplasmic calcium concentrations, altered transcription of genes influenced by FGFR signaling, and activation of the Janus kinase/signal transducers and activators of transcription (JAK/STAT) and MAPK pathways [77]. One notable inhibitor of FGFR signaling is the protein Sprouty2 (Spry2) which inhibits upstream activation of pathways that lead to activation of STATs and MAPKs. In the developing palate, FGFRs1–3 appear to play the most important role, though it does not appear that FGFR4 and 5 have been explicitly examined [79].

Studying the role of FGFRs in orofacial clefting is important because mutations in FGFRs1–3 are linked to syndromic cases of orofacial clefting, specifically with palate involvement [79,81]. Apert, Crouzan, Pfeiffer, Muenke, and Jackson-Weiss syndromes are all related to mutations in FGFRs and display a orofacial clefting phenotype in addition to craniosynostosis development [79]. Additionally, in up to 5% of non-syndromic cases of cleft lip with or without cleft palate, mutations in FGFRs have been identified as the causative factor [79]. Conditionally knocking out FGFR1 in mouse cranial derived neural crest cells, via utilization of Cre-recombinase driven by the Wnt1 promoter, results in clefting of the lip, primary, and secondary palate with a bilateral cleft palate encompassing both the anterior and posterior portions of the secondary palate [82]. Evaluation of cranial neural crest specific FGFR1 KO mouse embryos also reveals that the palatal shelves fail to elevate and that rugae fail to form completely [82]. Examination of gene expression at E10.5 and E14.5 in this model demonstrated increased BMP2 and BMP4 ligand expression, respectively, revealing that FGFR1 signaling alters BMP signaling, thereby further demonstrating the interconnectedness of pathways in palatogenesis [82]. Additionally, proliferation of the anterior epithelial cells and mesenchymal cells was found to be increased at E14.5, indicating that proliferation is not the cause of the clefting in the conditional FGFR1 KO models but that the etiology is restricted to failure of shelf elevation alone [82]. The lack of proliferative deficit in the conditional FGFR1 KO model may be due to the hypothesized redundant and compensatory function of FGFR1 and FGFR2 within the developing mammalian palate.

FGFR2 is divided into the epithelially restricted FGFR2b isoform and mesenchymally restricted FGFR2c isoform [79,80]. These alternatively spliced variants of FGFR2 possess differential binding affinity for the FGF ligands in which FGFR2b preferentially binds with FGF1, 3, 7, 10, 21, and 22 and FGFR2c preferentially binds with FGF1, 2, 4, 8, 21, and 23 [77]. This overlap in ligand specificity allows for FGFR2 signaling modulation based on exposure to a FGF ligand cocktail whose composition allows for precise control of FGFR2 signaling. Targeting epithelial expression of FGFR2 by utilizing a K14-Cre conditional knockout allows for the study of the phenotypic effects caused by the absence of FGFR2b [80]. Examination of this knockout model reveals that these mice die within 24 h of birth and are born with a multitude of defects, including cleft palate and agenesis of eyelids and bones of the lower limb [80]. Additionally, disruption of the FGF10/FGFR2b axis in palatogenesis reveals that, like the BMP4-SHH-BMP2 axis, any disruption involving SHH signaling between the mesenchyme and the epithelium may result in clefting [83]. The morphology of the cleft palate demonstrated that elevation of the shelves had occurred but that rugae were only rudimentarily formed [80]. Lack of rugae formation further supports that SHH signaling is affected in these mice where the FGF10/FGFR2b signaling pathway is disrupted [70,80]. Finally, examination of proliferation reveals decreased epithelial cell proliferation at E13.5 with overall morphology of the developing palatal shelves being smaller, indicative of a more widespread proliferative defect [80].

Unlike FGFR2b, in which absence may cause cleft palate, gain of function mutations in FGFR2c, such as those found in Crouzon and Pfeiffer syndromes, result in cleft palate [84]. Activating mutations are the best studied for FGFR2c, as inactivating mutations often results in embryonic lethality [85]. Clefting in this animal is restricted to the palate and results in a wide-open cleft secondary palate [84]. The affected mice also display increased ossification of the skeleton with premature cranial suture closure, craniosynostosis, thus leading to the hypothesis that the cleft palate found in these mice may be the result of a premature ossification of cranial neural crest derived mesenchymal osteoblasts in the palatal shelves [84]. If this premature ossification is occurring, then it may result in a decrease in the amount of proliferation that can occur prior to intramembranous ossification, resulting in morphologically normal shelves that do not contact in the midline. Global knockout of FGFR2b in mice presents with a cleft palate phenotype at birth similar to Msx1 knockouts and these mice die shortly after birth from numerous developmental abnormalities [86]. The most common craniosynostosis causing mutation of a FGFR is an overactivation mutation of FGFR3, called Muenke syndrome [81]. Structural abnormalities of the palate are present in 76% of Muenke syndrome patients but only 5% of patients with this syndrome are reported to have clefting of the lip and/or palate [81]. Lastly, mouse models in which Spry2 is absent display cleft palate in 18.8% of animals [87]. Proliferation is increased in Spry2 KO palatal mesenchymal cells possibly due to overexpression of Msx1 at E13.5 or overactivation of ERK1/2 following bFGF stimulus [87]. While mutations in FGFRs readily cause orofacial clefting, there is not much known about the role of FGF ligands in cleft development. One notable exception is FGF8 where inactivating mutations cause reduced shelf mesenchymal cell proliferation in the palatal shelf and delayed shelf elevation similar to the orofacial clefting observed in some cases of Kallman syndrome [88,89].

CCN2’s hallmark function is modulation of receptor signaling via modulation of ligand binding affinity; thus, CCN2 likely plays a role in regulating FGFR signaling during palatogenesis. Current data indicate that CCN2 is capable of interacting with FGFR2, FGFR3, FGF12, and FGF10 with high affinity [61]. Functional data demonstrate that addition of CCN2 potentiates binding of FGF2 and FGF4 to FGFR2c through a still unknown mechanism, and causes increased ERK1/2 phosphorylation following FGF2 stimulation in MC3T3-E1 cells [61]. Thus, in the absence of CCN2, FGFR2c signaling may be decreased due to a lack of CCN2 mediated FGFR2c signaling augmentation found under physiological conditions. Therefore, it is a likely possibility that the absence of CCN2 in the developing palate will cause dysregulation of the FGFR2b and FGFR2c signaling pathways resulting in a cleft palate phenotype similar to that observed in FGFR2b KO mice. Other studies, indicate that the CT domain (module 4) of CCN2 has inhibitory effects on FGF2 signaling since phosphorylation of ERK1/2, p38, and JNK MAPKs was decreased in the presence of CCN2 versus FGF2 alone [32]. If the inhibitory effects of the CT domain of CCN2 prevail, absence of CCN2 may lead to a clefting phenotype similar to activating mutations of FGFR2c as seen in the numerous craniosynostosis syndromes. Either scenario provides a basis for cleft palate development in the absence of CCN2.

#### 1.8.3. Epidermal Growth Factor (EGF)

EGF is the founding and prototypical ligand that activates the epidermal growth factor receptor (EGFR) [90]. EGFR is a 170 kDa transmembrane tyrosine kinase receptor that is a member of the ErbB family and is also known as ErbB1 or HER1 [91,92]. While EGF is the prototypical ligand, many ligands exist that activate EGFR, such as transforming growth factor alpha (TGF-α), amphiregulin, epiregulin, epigen, and heparin-binding EGF-like growth factor (HBEGF), although EGF and TGF-α are believed to be the most important ligands [90,91].

Ligand binding to the receptor initiates homo or hetero dimerization of the EGFR receptor with itself or other members of the ErbB family that results in phosphorylation of tyrosine residues in the cytoplasmic domains of each receptor [91]. The ligand that causes this dimerization determines which cytoplasmic tyrosine residues become phosphorylated and thereby may selectively initiate different signaling cascades from the same starting receptor [92]. These phosphorylated tyrosine residues then serve as attachment sites for cytoplasmic proteins to initiate signaling cascades [91]. Interestingly, receptor activation and downstream signaling may also occur in the absence ligands, through interaction with other ErbB family surface receptors, thus indicating that a complex level of regulation is necessary for proper EGFR signaling [91,92]. Downstream signaling pathways that are activated by EGFR dimerization include STATs, MAPKs, Src, and Ras pathways [91,92]. Activation of these pathways leads to altered gene transcription, which promotes cellular proliferation, adhesion, and migration [91,92]. EGF signaling governs cellular proliferation by enhancing cell cycle progression through the G1 phase possibly through activation of ERK1/2 [91,93]. EGFR may impact adhesion and migration of cells by altering the way the cells are capable of interacting with the surrounding ECM [94]. Additionally, EGFR likely influences the ability of cells to adhere and migrate by altering expression of focal adhesion kinase (FAK) and RhoA [93,95].

In the developing palate, EGF, TGF-α, and EGFR are mostly expressed in the proliferating medial edge epithelium (MEE) and the underlying mesenchyme of developing palates [96]. Therefore, it is not surprising, that when EGFR is absent in embryonic development, anomalies occur [97]. EGFR KO mice typically die within 1 week of birth due to respiratory problems and they also present with facial anomalies [97]. Characterization of these mice reveals hypognathia, lack of eyelids, and a cleft palate [97]. The clefting phenotype in EGFR KO mice is variable, ranging from submucosal clefts to wide open bilateral clefts of the secondary palate [97]. Gross morphology of the palatal shelves reveals underdeveloped shelves with normal rugae, suggesting a proliferative deficit and little or no impact on SHH signaling [97]. Aside from the proliferative deficit hypothesized to account for the smaller palatal shelves in some of the EGFR KO mice, a fusion deficit is observed in which an epithelial seam is retained at the midline of the developing palate [97]. This defect indicates that in the absence of EGFR the molecular pathways governing palatal fusion are negatively impacted. TGFβ-R3 signaling is one of these potential pathways and many of the downstream signaling components of the TGFβ-R3 pathway are also utilized by the EGFR signaling pathway, thus providing multiple opportunities for pathway disruption [55,56].

CCN2 has recently emerged as having EGFR activating abilities through interaction of the 4th domain of CCN2 with EGFR [24]. Stimulation with purified domain 4 causes phosphorylation of tyrosine residues 1068 and 1173 of EGFR [24]. This results in phosphorylation of ERK1/2 that can be inhibited by erlotinib, a clinically used EGFR inhibitor [24]. Thus, in the context of palatogenesis, CCN2 may act as a competitive agonist that may dampen, but not prevent, EGFR response through simultaneous expression in regions of the developing palate where EGFR ligands are also being produced. Through this interaction, CCN2 may be functioning as a regulator of EGFR signaling independent of EGFR or EGFR ligand expression. 

#### 1.8.4. Wnt Proteins

The Wnt protein family derives its name from a combination of wingless (wg), the Wnt1 ortholog in Drosophila melanogaster, and Int1 in mammals [98,99]. Currently 19 members have been identified in humans [98,99]. Wnt proteins are secreted lipid-modified glycoproteins that govern patterning of the body and control cellular proliferation, migration, differentiation, tissue homeostasis, and stem cell maintenance based on the concentration gradient that develops as Wnt proteins diffuse away from the producing cells [98,99]. This concentration gradient allows for spatial regulation of Wnt responsive genes in cells expressing the Wnt G-protein coupled receptor Frizzled (Fz) and the low-density lipoprotein related receptors 5 and 6 (LRP5 and 6) [98,99,100]. Activation of Fz by Wnt binding leads to activation of canonical and non-canonical Wnt signaling, depending on the Wnt ligand bound [99]. Activation of the canonical pathway results in β-catenin translocation to the nucleus while activation of the non-canonical pathway results in activation of the c-Jun N-terminal kinase (JNK) MAPK, RhoA, Rac, and protein kinase c (PKC) [99,100]. Wnts 1, 3a, and 8 are known to activate the canonical Wnt/β-catenin pathway, though it is likely other Wnt proteins activate this pathway as well [99,100].

In the absence of Wnt activation of Fz, a complex of proteins that include disheveled, GSK-3β, and casein kinase 1α, assemble just beneath the cell membrane, resulting in the phosphorylation of β-catenin causing phosphor-β-catenin to be targeted for ubiquitination by E3 ubiquitin ligase and proteasomal degradation [98,99]. Alternatively, Wnt activation of Fz results in phosphorylation of disheveled, which prevents assembly of the protein complex responsible for phosphorylation of β-catenin, resulting in less β-catenin degradation [98,99]. Prevention of β-catenin degradation allows the cytoplasmic level of β-catenin to increase, causing increased translocation of β-catenin into the nucleus, which allows interaction with the TCF/LEF transcription factors leading to enhanced canonical Wnt target gene transcription [99]. Non-canonical activation is facilitated through binding of Wnts 4, 5a, and 11, though other members likely are involved here as well [100]. Similar to the canonical pathway, binding of Wnts activates Fz and disheveled in the non-canonical pathway but then activates RhoA or Rac leading to downstream JNK activation to impact cytoskeletal structure and JNK translocation into the nucleus to affect gene transcription [99]. Similarly, activation of Rho family proteins could lead to activation of YAP/transcriptional co-activator with PDZ-binding motif (YAP/TAZ) leading to transcription of genes known to inhibit canonical Wnt signaling, such as BMP4 and CCN2 [101]. Likewise, disheveled may also stimulation phospholipase C (PLC) leading to diacylglycerol (DAG) and inositol triphosphate (IP3) production to cause a cytoplasmic calcium concentration increase, leading to inhibition of β-catenin and activation of NF-κB responsive genes [99].

**Figure 4:**
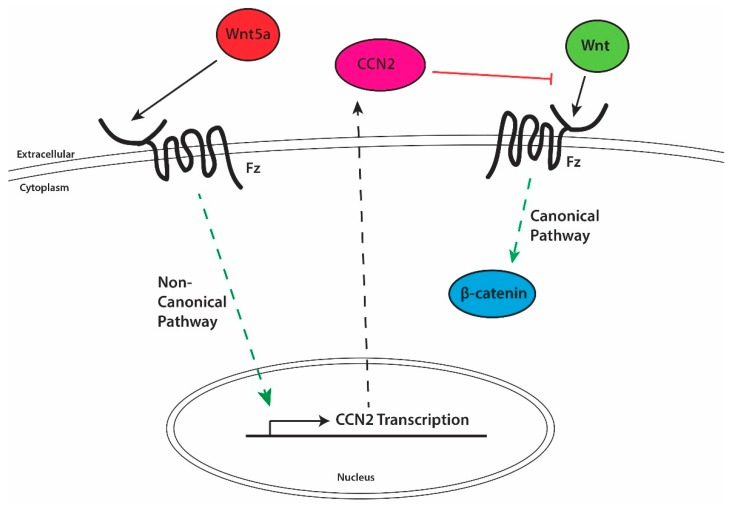
The role of CCN2 in regulation of LRP5/6 dependent Wnt signaling. Wnt5a signals through Fz to induce CCN2 transcription via the non-canonical Wnt pathway. Once secreted into the extracellular environment, CCN2 then inhibits other Wnt signaling to reduce canonical Wnt signaling. Absence of either Wnt5a or CCN2 results in possible over activation of canonical Wnt signaling.

In the context of palate development, 12 of the 19 known human Wnts are detectable at the mRNA level in the developing palate [100]. Within the developing palate, Wnt gene expression is temporally and spatially specific to individual Wnt genes [100]. Wnt4, 10a, and 10b are restricted to the palatal epithelium, while Wnt2 and Wnt16 are expressed within the mesenchyme [100]. Wnt10a and 10b are only observed from E13.5 to E14.5, while Wnt4, 2, and 16 are seen at E12.5 through E14.5 [100]. Wnt4, 5a, 10a, 10b, and 11 expression is restricted to the epithelium and is seen on both the nasal and oral epithelial edges [72,100,102]. Absence of Wnt5a during palatogenesis results in a bilateral cleft palate only that extends the entire length of the secondary palate [72]. Histological examination of E13.5 coronal sections reveals that the anterior palatal shelves of these knockouts mice experience increased proliferation in the mesenchymal tissue concentrated at the nasal side of the palatal shelf, while posterior palatal shelves show reduced proliferation [72]. This increased nasal sided proliferation in the anterior palatal shelf results in palatal shelve that are decreased in length along the x-axis in a coronal section but increased in length along the y-axis. In addition, despite normal anterior shelf elevation, these shelves also fail to grow toward the midline and converge due to the misdirected proliferation at E14.5 [72]. As mentioned above, Wnt proteins influence development by creating a gradient away from the Wnt secreting cell, thus, the abnormal shape displayed in the Wnt5a KO mouse model of cleft palate only and the spatially separated mesenchymal cell proliferative rates may be explained by abnormal Wnt gradients. Conversely, the lack of Wnt5a responsive gene expression could impact other spatially regulated proliferative factors such as BMPs. In fact, in-situ hybridization of suspected dysregulated genes reveals significantly decreased BMP2 and 4 expression as well as SHH expression away from the normal location when Wnt5a signaling is missing in palatogenesis [72]. As mentioned above, Wnt5a causes transcription of CCN2, a canonical Wnt pathway inhibitor (Figure 4) [101]. CCN2 likely inhibits this pathway through its known interaction with LRP5 and 6, known co-receptors involved in Wnt signaling [23]. Thus, any Wnt that utilizes LRP5 or LRP6 as co-receptors has the potential to be inhibited by CCN2 (Figure 4). These include but are not limited to Wnt1, Wnt2, Wnt3, Wnt3a, Wnt6, Wnt7a, Wnt7b, Wnt9b, Wnt10a, and Wnt10b [103,104]. In the absence of CCN2, one would predict that canonical Wnt signaling is increased, leading to excessive β-catenin responsive gene transcription, while changes in the non-canonical pathways are more difficult to predict (Figure 4). Both pathways warrant further analysis in the developing palate of the CCN2 KO embryos.

#### 1.8.5. Transforming Growth Factor β (TGF-β)

The TGF-β pathway, specifically TGF-β1, is the best-studied regulator of CCN2 expression; however, TGF-βs are involved in regulating a myriad of cellular and developmental processes, including palatogenesis. The TGF-β ligands are expressed in the developing palatal shelves as early as E13.5 where expression of mRNAs for TGF-β1 and 3 are observed in the MEE and TGF-β2 is observed in the mesenchyme underneath the epithelium [105]. TGF-β1 and 3 expression is restricted to the epithelium of the developing palate until post-fusion when their expression shifts to the mesenchyme to govern TGF-β1 mediated ossification of the palatine bones and nasal processes and TGF-β3 mediated chondrification of the nasal septum and anterior secondary palate [105]. TGF-β2 expression is seen in the mesenchyme of the developing palate throughout palatogenesis but appears to be highest in the area just under the epithelium and diffuses as a gradient deep to the epithelium [105]. Upon contact of the shelves at the midline and during the process of fusion, TGF-β2 expression increases in the mesenchyme on both sides of the epithelial seam with higher concentrations seen at the nasal side of the shelves [105]. TGF-β1 KO mice do not display cleft palate phenotypes, however, TGF-β2 and 3 KO mice show clefting involving defects in growth and fusion, respectively [106,107]. TGF-β2 KO mice die due to hypoxia owing to insufficient pulmonary function, similar to the EGFR, FGFR2b, and CCN2 KO models [106]. Some (23%) of the TGF-β2 KO mice exhibit clefting of the secondary palate that extends the entire length of the secondary palate but does not involve the primary palate or upper lip [106]. Histological examination of TGF-β2 KO mice reveals failure of palatal shelf elevation at E18.5, indicating that failure of elevation is not merely delayed but does not occur at all [106]. TGF-β3 KO mice also die shortly after birth, due to lack of feeding owing to inability to suckle, and show cleft palate with the defect originating in failure of shelf fusion with 100% penetrance [108,109,110]. The degree of clefting in the TGF-β3 KO mice is variable because the lack of fusion allows for the midline seam to be pulled apart easily as the head of the developing fetus grows larger.

Like the ligands, the TGF-β receptors also have links to cleft palate [55,56,111,112]. Global knockout of TGF-βR1 is embryonic lethal, thus to study the role of TGF-βR1 in palatogenesis, conditional knockouts are used [111]. In epithelial specific knockouts, TGF-βR1 displays 100% penetrance of cleft soft palate due to a fusion deficit [111]. At the molecular level, the cause is believed to be a lack of apoptotic response of the MEE cells following midline contact of the palatal shelves and thus as the head grows the shelves separate to form a cleft palate [111]. In cranial neural crest cell specific TGF-βR1 KO mice, much more severe facial defects are present, including cleft lip and cleft palate running the entire length of the palate [111]. Like TGF-βR1, TGF-βR2 global knockout mice also die early in embryogenesis, around E10.5, thereby facilitating the need for conditional knockouts in order to study TGF-βR2 in palatogenesis [112]. Cranial neural crest cell specific knockout of TGF-βR2 yields a cleft secondary palate without primary palate involvement [112]. Characterization of the defect reveals palatal shelves that have elevated normally but fail to grow and meet in the midline [112]. Interestingly, when apposed artificially in an organ culture system, all pairs of shelves fuse normally, indicating that the fusion mechanism is functioning normally [112]. Examination of proliferation reveals that the TGF-βR2 KO cells suffer a proliferative defect starting 14.5 due to decreased Cyclin D1 expression, indicating that there may be an issue progressing through the G1/S cell cycle checkpoint in these animals [112]. The TGF-βR3 global KO is not embryonic lethal and TGF-βR3 KO mice have a proliferative defect that results in smaller palatal shelves that fail to elevate similar to TGF-βR1 and R2 KO mice [56]. When the free edges of the palatal shelves are apposed in organ culture, unlike TGF-βR2, a fusion defect is evident, indicating that TGF-βR3 has a vital role in the fusion mechanics of the palatal shelves as well as mesenchymal proliferation [56]. Further examination of the effects of TGF-βR3 KO on TGF-β/BMP ligands and receptors reveals that TGF-β2 and 3, TGF-βR1 and 2, BMP2 and 4, and BMPR1a mRNA levels are all decreased in palatogenesis [56]. TGF-β1 signaling induces CCN2 production, while CCN2 itself potentiates TGF-β signaling through a process thought to involve increasing affinity of the receptor for the ligand [29,46]. Much like the absence of the TGF-β ligands and receptors, absence of CCN2 likely reduces TGF-β signaling because of a reduction in ligand-receptor affinity. The morphology of the cleft palate in the CCN2 KO model demonstrates similarities to that of the TGF-β2, TGF-βR1, TGF-βR2, and BMPR1a knockouts, therefore warranting additional studies to examine how the absence of CCN2 effects TGF-β/BMP ligands and receptors in the developing palate.

### 1.9. Future Research Directions

**Figure 5:**
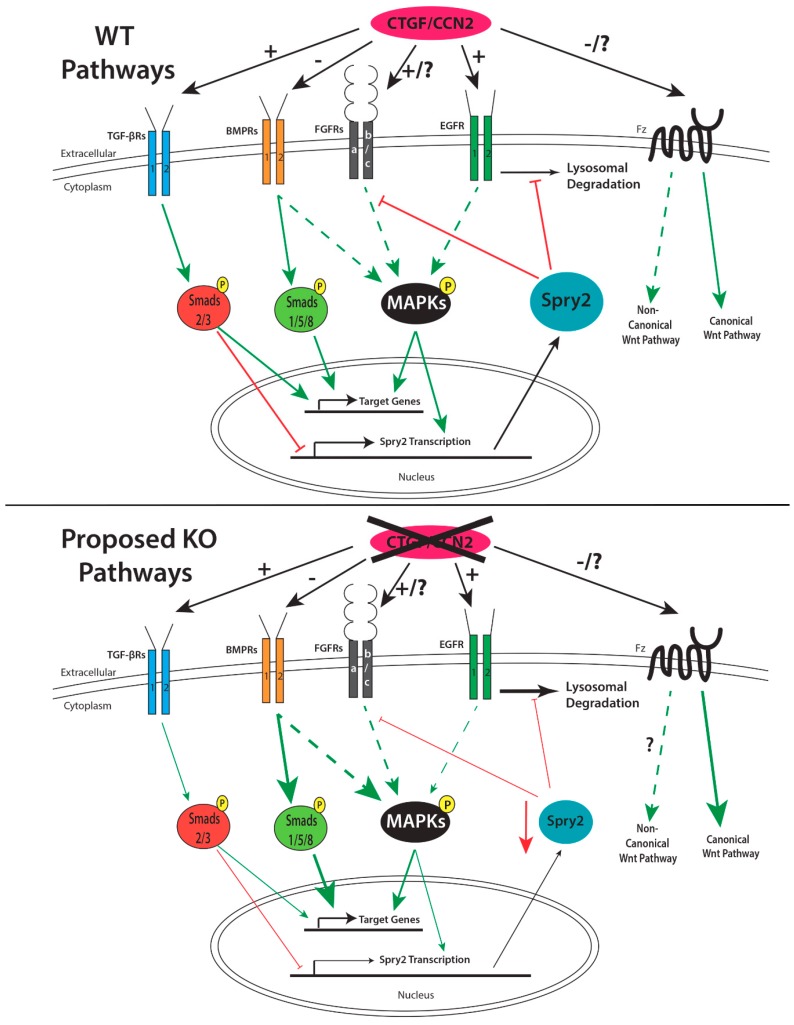
Summary of the role of CCN2 in palate developmental pathways in the WT environment and proposed effects in the CCN2 KO setting. In the absence of CCN2, some signaling pathways (e.g., TGF-β, EGF, and FGF) may be reduced, while other pathways (e.g., BMP and the canonical Wnt pathway) may be augmented. On the other hand, if the FGF pathway inhibitor SPRY2 is decreased, this could result in increased FGFR and EGFR signaling. In the absence of CCN2, the potential for dramatically altered expression of downstream target genes for each of these signaling pathways exists and warrants further exploration in the developing palatal shelves of CCN2 KO embryos.

Normal palate development is a complex process that involves numerous growth factors, their receptors and intracellular signaling pathways. Clefting of the palate can result from dysregulation in the precise timing and/or localization of expression of any of these ligands, receptors or signaling pathways, some of which have been previously identified. Recently, an indispensable role for CCN2 in palate development has been documented, and in the absence of CCN2, mice exhibit clefting of the secondary palate with 100% penetrance. In addition to a direct role for CCN2 in regulating various cellular processes necessary for palate development, CCN2 also interacts with a number of other factors known to regulate palate development (Figure 5).

Future studies directed at elucidating the mechanisms of action of CCN2 in palatogenesis should include the use of conditional knockout mice (in vivo) and culture of cells obtained from these mice (ex vivo). The expression of other factors shown to interact with CCN2 should be examined in the aberrant palatal shelves from CCN2 KO mice, and isolation of mesenchymal cells from these KO mice would allow for examination of signaling pathways induced by these related factors in cell culture experiments. Generating conditional Wnt1-Cre CCN2 knockout mice (Wnt1-CCN2 cKO) would allow for examination of the role of CCN2 expression, specifically in cranial neural crest-derived mesenchymal cells during palate development. If the Wnt1-CCN2 cKO exhibits a cleft palate, this model would allow one to determine whether abrogation of the tongue/mandible phenotype observed in the global CCN2 KO has any effect on palate shelf formation, elevation or fusion. Furthermore, the generating conditional KO mice using odd-skipped related transcription factor-1 (Osr1)- and Osr2-Cre mice would allow for examination of the role of CCN2 in mediolateral patterning of the palatal shelf. These in vivo and ex vivo approaches are likely to enhance our understanding of the precise mechanisms involving CCN2 and other factors with which it interacts during palatogenesis.
